# Smartphone-Based Portable Bioluminescence Imaging System Enabling Observation at Various Scales from Whole Mouse Body to Organelle

**DOI:** 10.3390/s20247166

**Published:** 2020-12-14

**Authors:** Mitsuru Hattori, Sumito Shirane, Tomoki Matsuda, Kuniaki Nagayama, Takeharu Nagai

**Affiliations:** 1 The Institute of Scientific and Industrial Research (SANKEN), Osaka University, 8-1 Mihogaoka, Ibaraki 567-0047, Japan; 8tr@sanken.osaka-u.ac.jp (M.H.); tmatsuda@sanken.osaka-u.ac.jp (T.M.); 2 Science Communication Research Institute, Lions Plaza Minami Ohta 417, 1-11-13 Minami-Ku, Yokohama 232-0006, Japan; s.shirane@scri.co.jp (S.S.); nagayama@nips.ac.jp (K.N.); 3 N-EM Laboratories Inc., Tokyo Tech Yokohama Venture Plaza E109, 4259-3 Nagatsuta, Yokohama 226-8510, Japan

**Keywords:** bioluminescence, smartphone, microscope, imaging, personal, portable

## Abstract

Current smartphones equipped with high-sensitivity and high-resolution sensors in the camera can respond to the needs of low-light imaging, streaming acquisition, targets of various scales, etc. Therefore, a smartphone has great potential as an imaging device even in the scientific field and has already been introduced into biomolecular imaging using fluorescence tags. However, owing to the necessity of an excitation light source, fluorescence methods impair its mobility. Bioluminescence does not require illumination; therefore, imaging with a smartphone camera is compact and requires minimal devices, thus making it suitable for personal and portable imaging devices. Here, we report smartphone-based methods to observe biological targets in various scales using bioluminescence. In particular, we demonstrate, for the first time, that bioluminescence can be observed in an organelle in a single living cell using a smartphone camera by attaching a detachable objective lens. Through capturing color changes with the camera, changes in the amount of target molecules was detected using bioluminescent indicators. The combination of bioluminescence and a mobile phone makes possible a compact imaging system without an external light source and expands the potential of portable devices.

## 1. Introduction

Given the recent development in camera performance in smartphones, such as sensitivity and resolution, their application as an analytical tool in the scientific field cannot be ignored. A complementary metal-oxide semiconductor (CMOS) image sensor is the main detector for a smartphone’s built-in camera, which currently has high pixel counts over 10 mega [1,2]. Such numbers already exceed those of cameras conventionally used in scientific research. In addition, it can support various situations such as the requirement of high-sensitivity, time-lapse shooting, stream acquisition, and long exposure. Because of these strong capabilities, several smartphone-based methods for analyzing biological phenomena have been reported [3,4,5,6,7]. Fluorescent tags are the tools to perform imaging in combination with smartphones, and various fluorescently labeled targets have been detected by a smartphone camera [8,9,10]. In addition, the combination of a smartphone camera with an optical attachment, a fluorescence microscope, has been developed that can observe specific targets in cells [11,12,13]. At present, fluorescence detection with a smartphone camera is also applied to super-resolution fluorescence imaging to detect single molecules in cells [14].

Another aspect of smartphone utility is its mobility. A smartphone comprises the basic components of an imaging device including a sensor, display, and computer. Moreover, it is equipped with a mobile battery and can function anywhere. The captured data can also be saved to a cloud server via Wi-Fi. Therefore, these mobile platforms greatly expand the potential for scientific research, which opens up possibilities for applications that were previously unthinkable. For the purpose of this mobility, however, a set of an external light source and filters should always be attached to a smartphone camera when observing fluorescence. Such additional devices impair the portability of smartphones.

To mitigate this limitation, we focused on the use of bioluminescence for biological detection. Bioluminescence is generated by a bioluminescent protein such as luciferase. Luciferase produces bioluminescence by an enzymatic chemical reaction with a substrate called luciferin; therefore, we can obtain the luminescence signal without any external light source. In considering its application to smartphones, the property of bioluminescence that does not require the excitation light leads to the realization of a compact imaging device. Several methods for measuring bioluminescent indicators using smartphones have been proposed [15,16]. However, there are no applications to bioluminescence imaging at the cellular level.

Herein, we attempted to establish bioluminescence imaging methods using a smartphone camera. As bioluminescent probes, we selected a color palette of enhanced Nano-lanterns (eNLs) [17] that are chimeric proteins of NanoLuc [18] with color variants of fluorescent proteins. With this simple method, we could measure various scale targets from whole mouse body to organelle.

## 2. Materials and Methods

### 2.1. Smartphone and Detachable Objective Lens

A Samsung Galaxy S8+ (out camera: 1220 × 10^4^ pixels, pixel size: 1.44 µm, focal length: 4.2 mm, F-score: 1.7) was used for all smartphone-based analyses. The optical or digital zoom was not activated. The body of the equipment attached to the smartphone and its shading case were made of acrylonitrile-butadiene styrene (ABS) resin using a three-dimensional printer. A compound lens consisting of a spherical lens was purchased from a Japanese mobile-lens manufacturing company. Information on the specifications of the lens were provided by the manufacturer as follows: magnification, 180×; focal length, 2.9 mm; numerical aperture (N.A.), 0.25; working distance, 3.8 mm; theoretical resolution, 1.44 µm. The spatial resolution was calculated from the full width at half maximum to be 2.1 µm (Supplementary Materials Figure S2). The equipment was attached to the smartphone using a double-sided tape.

For brightfield images, room light or LED light was used as the light source. Images for measuring point spread function were taken by dark-field observation with fluorescent beads (diameter: 500 nm) and an LED light source. All photos were managed by the pre-set camera software in Android OS (manual mode, ISO800; white balance: 5000–5500 K).

### 2.2. Gene Materials

The cDNA of NanoLuc was provided by Promega and was inserted into the pcDNA3vector (Invitrogen) for expression in mammalian cells. The pcDNA3 coding for the five color variants of enhanced Nano-lantern (eNL, cyan, green, yellow, orange, and red) was the same as registered in Addgene (ID: 85199, 85200, 85201, 85202, and 85203). Large-scale plasmid DNA was obtained from 200 mL of Luria-Bertani (LB)-liquid culture by using the alkaline– sodium dodecyl sulfate (SDS) lysis method, PEG8000 precipitation, and two rounds of phenol/chloroform extraction. The cDNAs of CalfluxVTN [19], GeNL(Ca^2+^)_520 [17], and GeNL(Ca^2+^)_520-H2B [20] were the same as in each reference. For mammalian cell expression, these cDNAs were amplified by PCR using specific primers and inserted into pcDNA3 using *Bam*HI and *Eco*RI sites.

### 2.3. Live Cell Imaging

The HeLa (RIKEN BRC) cells were cultured on a 24-well polystyrene plate in Dulbecco’s modified Eagle’s medium (DMEM) supplemented with 10% fetal bovine serum (FBS). Cells under 50% confluency were transfected with pcDNA3 vectors coding each bioluminescent protein using Polyethylenimine Max (Polysciences, Inc.). Cells were re-cultured in 35 mm glass-bottom dishes after 20 h and incubated for 24 h. The medium was replaced with Hank’s Balanced Salt Solution (HBSS)(–) supplemented with 20 µM Furimazine (Promega) as substrate for NanoLuc and enhanced Nano-lanterns. For Ca^2+^ imaging, phenol red-free DMEM/F12 supplemented with 20 µM Furimazine was used. Fluctuation of Ca^2+^ in cells was induced with 10 µM histamine. Each image was taken using a smartphone camera with the attached objective lens. The exposure time was 10 s/frame. The colors in the original images were separated using Metamorph software and measured for intensity. The HeLa cells stably expressing yellow eNL were established by screening with G418 (Sigma). The localization of GeNL(Ca^2+^)_H2B was confirmed with a fluorescence microscope, IX-83 (Olympus). Hoechst33342 (Molecular Probes) was used as a nucleus marker.

### 2.4. Mouse Imaging

The HeLa cells transiently expressing cyan and red eNL (1 × 10^5^ cells) were suspended in 100 µL of PBS and injected under the skin of a BALB/c nude mouse (female, 5 weeks old, 16 g body weight). One hundred microliters of PBS with 20 µM Furimazine was injected in the same region.

## 3. Results

**Figure 1:**
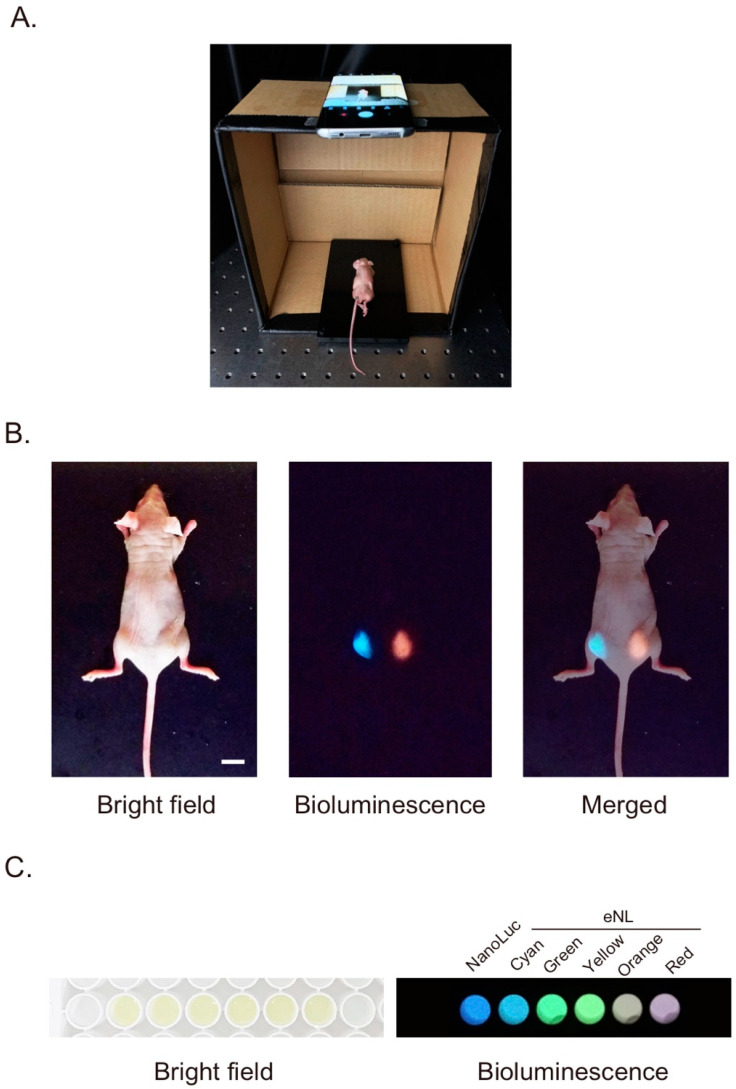
Simultaneous multiple wavelength bioluminescence imaging using a smartphone camera. (**A**) Imaging setup: a cardboard box was used as a stand for the smartphone. (**B**) Whole body imaging of a mouse. HeLa cells transiently expressing cyan eNL or red eNL were injected subcutaneously into the mouse. The bioluminescence image was taken in a dark room. Scale bar, 10 mm. (**C**) Wide-field bioluminescence image of cultured cells on a 96-well plate. HeLa cells transiently expressing NanoLuc or each color variant of eNL were cultured on a 96-well plate. Bright field and bioluminescence images were taken by adding the substrate.

First, we tried to perform bioluminescence imaging of a living body. In the application of bioluminescence imaging to tissues and living organisms, the advantages over fluorescence were even more pronounced. There are many considerations when using fluorescent markers for in vivo imaging. It is difficult to irradiate the markers existing inside of a living body [21]. Furthermore, biological samples have several sources for generating autofluorescence [22]. Bioluminescent probes can address those concerns owing to their spontaneous emissions. Especially, using the color palette of eNLs [17], different targets can be distinguished by their perspective bioluminescence colors. The HeLa cells (1 × 10^5^) transiently expressing cyan and red color variant of eNLs were injected subcutaneously into a mouse, and the substrate was added to the same area. Bioluminescence imaging generally needs to be performed in a dark environment to prevent external light leakage; therefore, we set the mouse on a hand-made smartphone stand and took the bioluminescence image in the dark (Figure 1A). As a result, different emission colors could be simultaneously detected by the smartphone camera without using any filter (Figure 1B). In a conventional in vivo imaging system, a cooled charge coupled device (CCD) or electron multiplying CCD (EM-CCD) image sensor was mainly used for high-sensitive imaging. However, most of these are monochromatic image sensors that cannot separate the wavelengths by themselves; hence, it was necessary to take an image of each wavelength by alternating optical filters. In contrast, because the camera mounted on smartphones is a color camera that identifies colors by the intensity balance of red, green, and blue, bioluminescence at different wavelengths can be detected simultaneously. Therefore, the smartphone imaging method does not need additional filters, resulting in a compact design for the whole device.

The property of simultaneously detecting multi-color bioluminescence may also be useful for the biological assay or diagnosis of multiple samples using a plate reader. In this case, a time lag occurred in the measurement between the wells because the bioluminescence from each well of the plate was sequentially detected by a photo multiplier tube (PMT). To avoid errors due to the time lag, a method was proposed for taking the whole well plate image with an EM-CCD [23]. Even with this method, if multiple wavelengths needed to be detected, a time lag occurred depending on the time required for the replacement of the optical filter. Therefore, we attempted to perform filter-free multiple wavelength imaging of bioluminescent samples on a 96-well plate using a smartphone camera (Figure 1C). We cultured HeLa cells (0.5 × 10^5^ per well) transiently expressing NanoLuc or any of the five color variants of eNL, cyan, green, yellow, orange, and red, on a 96-well plate and took bioluminescence images in each well. Owing to the one-shot acquisition of multicolor images, the throughput of data acquisition could be dramatically improved.

**Figure 2:**
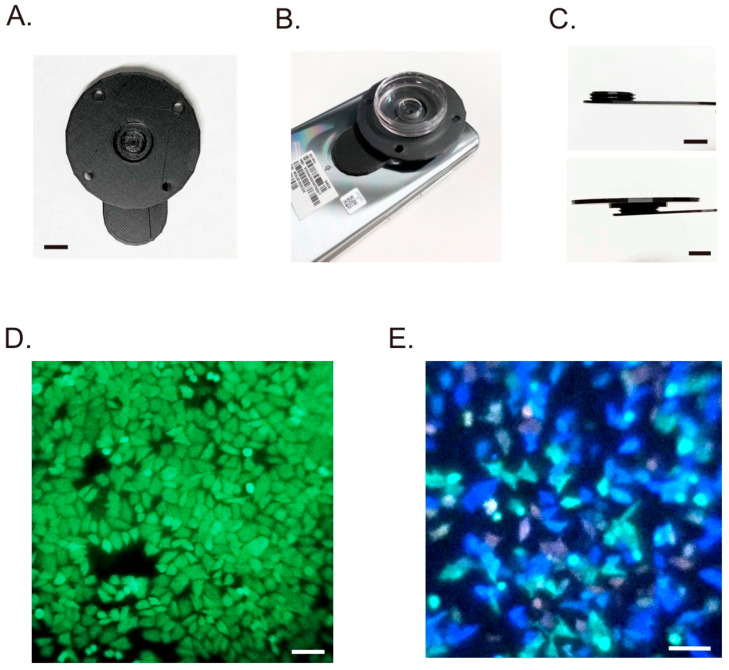
Bioluminescence imaging using the smartphone microscope. (**A**) Overview of the detachable objective lens. Images were taken from the front. Scale bar, 10 µm. (**B**) Attachment of the lens to the outer camera of the smartphone. (**C**) Side views of the equipment. The lens part had a screw (top), and the pedestal part was connected (bottom). The focus was adjusted by rotation of the pedestal. Scale bar, 10 mm. (**D**) Cell population imaging using the smartphone microscope. HeLa cells stably expressing yellow eNL were cultured on a dish and observed. Scale bar, 100 µm. (**E**) Population imaging of HeLa cells transiently expressing either NanoLuc or any colors of eNL (cyan, green, yellow, orange, or red). Scale bar, 100 µm.

Next, we examined whether the application extended to single-cell bioluminescence imaging. To take images with a spatial resolution at the single-cell level, we developed detachable optical equipment for a smartphone camera. Assuming the use of a 35 mm glass-bottom dish, the equipment comprised two parts: an objective lens and a pedestal (Figure 2A and Figure S1A). The distance between the lens and the bottom of the dish can be adjusted by rotating the pedestal (Figure S1B). The equipment is installed to the inner or outer region of the camera of the smartphone using a double-sided tape (Figure 2B). A shading cover for the culture dish can be used to capture images in places with light (Figure 2C). Based on the specification of the objective lens, the theoretical spatial resolution was 1.44 µm at 590 µm fluorescence. Optical aberration was not considered; therefore, effective resolution when mounted on a smartphone camera was verified using a micrometer. The objective lens can easily recognize the minimum distance between scales of 10 µm (Figure S2A,B). Therefore, it was estimated to have sufficient effective spatial resolution to distinguish individual cells. In the image of the micrometer, some distortions were observed at the edge of the field of view. To assess the distortion at each point of view, we analyzed the point spread function using fluorescent beads (Figure S2C). Compared to the center region, the midpoint of the field exhibited a larger point spread. Therefore, we chose the central area to obtain a more accurate image.

Owing to the effective resolution, the smartphone microscope has the potential to detect bioluminescence from an individual cell. To demonstrate this, HeLa cells stably expressing the yellow color variant of eNL were cultured on a dish, and an image was taken after adding the substrate (Figure 2D). The result revealed that each cell could be clearly distinguished at the cell boundary. Next, the bioluminescence color discrimination ability of the smartphone microscope was examined. We performed bioluminescence imaging of the population of HeLa cells transiently expressing NanoLuc or any of the five color variants of eNL (Figure 2E). The emission peaks of the six bioluminescent proteins ranged from 400 to 700 nm [17], and the smartphone microscope could simultaneously detect each colored cell. Therefore, we established a filter-free, multi-color bioluminescence single cell imaging technique using a smartphone camera.

**Figure 3:**
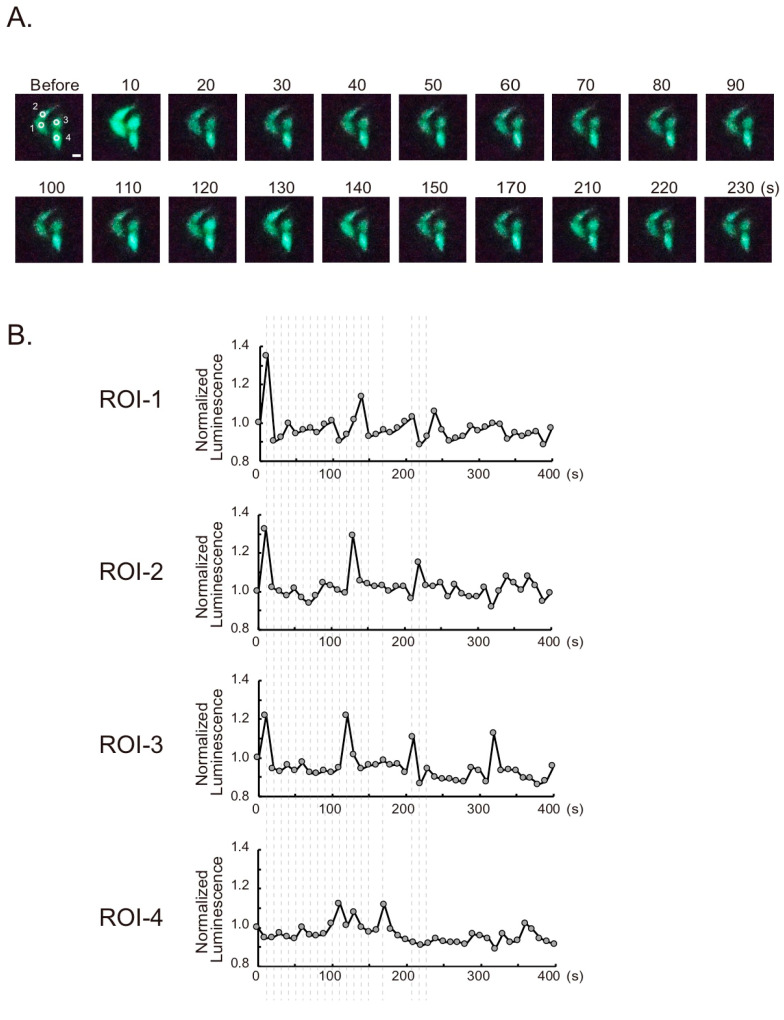
Ca^2+^ imaging with GeNL(Ca^2+^)_520 in HeLa cells based on the smartphone microscope. (**A**) Time-lapse bioluminescence images of GeNL(Ca^2+^)_520 in cytosol. Cells were stimulated by histamine. The numbers indicate the elapsed time after stimulation. Scale bar, 20 µm. (**B**) Time-course variations of bioluminescence intensity in cells. Regions of interest (ROIs) for the measurement are shown as white circles in A. The *x*-axis shows the elapsed time after the stimulation (at 0). Broken lines indicate the time points of selected images in A.

For cultured cells expressing Ca^2+^ indicators, we detected the change in bioluminescence using the smartphone microscope. The GeNL(Ca^2+^)_520 is an intentiometric Ca^2+^ indicator based on eNL [17]. Some of the cells showed a histamine-dependent transient increase in bioluminescence (Figure 3A,B). Several tens of seconds after the stimulation, the emission intensity returned to the base line, and a spike in emission was observed, which is a typical response seen during Ca^2+^ oscillation. The differences in the timing of Ca^2+^ spikes between the cells were also observed by the smartphone camera. Therefore, the time resolution and sensitivity of the microscope were sufficient to observe the cellular events by bioluminescence. For observing a faster phenomenon, the exposure time can be shortened, considering the balance with the bioluminescence intensity.

**Figure 4:**
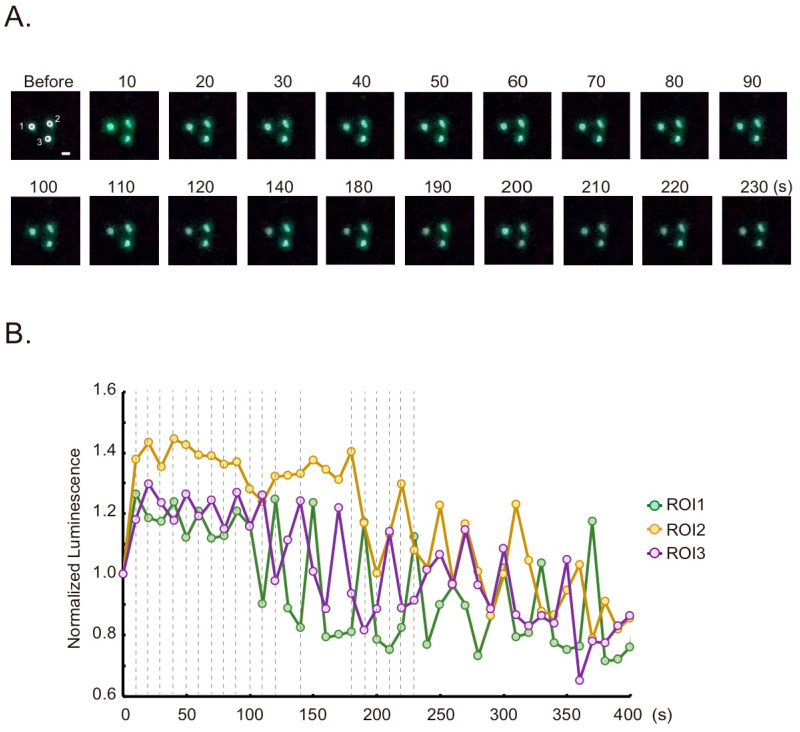
Ca^2+^ imaging in the nucleus with GeNL(Ca^2+^)_520-H2B based on the smartphone microscope. (**A**) Time-lapse bioluminescence images of GeNL(Ca^2+^)_520-H2B in the nucleus. HeLa cells transiently expressing the indicator were stimulated by histamine. The numbers indicate the elapsed time after stimulation. Scale bar, 20 µm. (**B**) Time-course variation of bioluminescence intensity in the nuclei. Regions of interest (ROIs) for the measurement are shown as white circles in A. *X*-axis shows the elapsed time after the stimulation (at 0). Broken lines indicate the time points of selected images in A.

Tagged with the nuclear target signal peptide, histone 2B, GeNL(Ca^2+^)_520 can be used for monitoring Ca^2+^ changes in the nucleus (Figure S3, GeNL(Ca^2+^)_520-H2B). Luminescence was observed in the nucleus as a green color (Figure 4A, “Before”). Upon histamine stimulation, the bioluminescence oscillation was observed in each nucleus (Figure 4B). The difference in Ca^2+^ changes between the cytoplasm and nucleus has been discussed several times, and one report using another Ca^2+^ indicator showed no significant difference [24]. Our results, including Figure 3, agree with these reports.

Therefore, the combination of a smartphone camera, a detachable objective lens, and the bioluminescence indicator allow for real-time imaging of Ca^2 +^ dynamics within cellular organelles.

**Figure 5:**
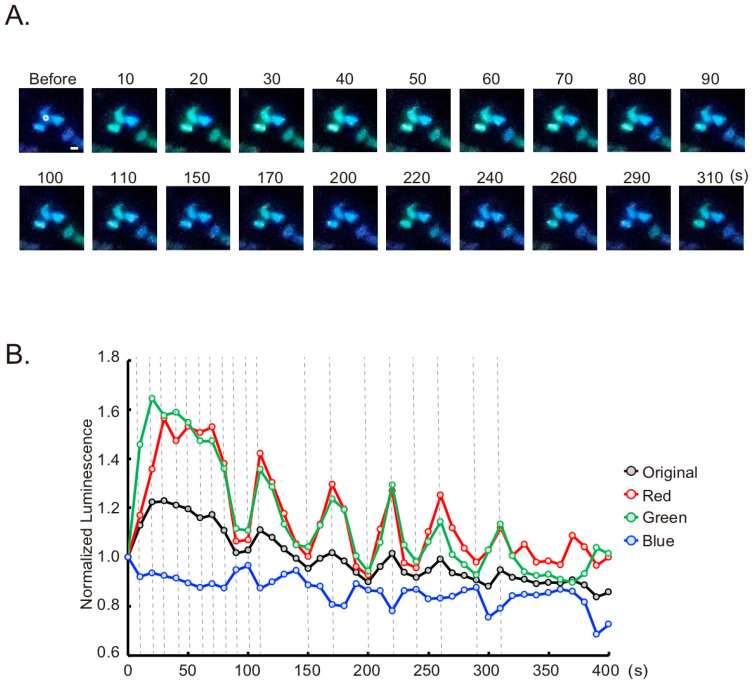
Ca^2+^ imaging with CalfluxVTN in HeLa cells based on the smartphone microscope. (**A**) Time-lapse bioluminescence images of CalfluxVTN in cytosol. Cells were stimulated by histamine. The numbers indicate the elapsed time after stimulation. Scale bar, 20 µm. (**B**) Time-course variation of bioluminescence intensity in cells. The intensity was measured for each color image separated from the original (Figure S4). Region of interest (ROI) for the measurement is shown as the white circle in A. The *x*-axis shows the elapsed time after the stimulation (at 0). Broken lines indicate the time points of selected images in A.

We also detected changes in emission color of the other indicator using the smartphone microscope. CalfluxVTN is a ratiometric Ca^2+^ indicator which was based on NanoLuc and a fluorescent protein, Venus [19]. Through the smartphone camera images, CalfluxVTN showed blue bioluminescence in the cytosol (Figure 5A, “Before”). Upon histamine stimulation, the color changed to green, repeating the sequence of blue and green, which reflects Ca^2+^ oscillation (Figure 5A,B and Figure S5). By using a smartphone camera, it is possible to detect the signal of an indicator as the bioluminescent color change without using a spectral filter.

## 4. Discussions

We developed smartphone-based methods for bioluminescence imaging at various scales. Especially for single cell imaging, this imaging system was able to detect the Ca^2+^ dynamics in cells through bioluminescence. In this study, we used an outer camera of the smartphone for observation. The developed objective lens can also be attached to the inside camera according to the observation target. Furthermore, we can also connect the smartphone to other devices to acquire the images remotely.

For simultaneous multiple wavelength imaging as shown in Figure 2E, it is necessary to consider chromatic aberrations that affect the image quality. To eliminate this influence on the image, a lens with an optical system for compensation of chromatic aberrations can be combined with the system. In addition, since smartphones usually corrects the images automatically, they do not always accurately represent the true light emitted by the object. It is important to establish a smartphone app that allows manual adjustments of the image acquisition setting including this point.

The compact size of the smartphone microscope is useful not only for its portability but also for applications in space. Stress and damage to cells in weightless space are important for study in space exploration [25,26,27]. Because of the limited space available in a spacecraft and space stations, it is essential that the measurement equipment used are as compact as possible with minimal components. Smartphone-sized detectors are suitable for this purpose, and the bioluminescent techniques are convenient in such extreme environments.

Observation of plants is also an important application of bioluminescence imaging using smartphones. Plant cells are often light-sensitive, and observation with fluorescent labels can affect their activity owing to the intense illumination by the excitation light. Recently, the development of autoluminescent plants [28] has made it possible to observe plants without the need for the excitation of light or the addition of substrates. The smartphone microscope, which can easily observe the bioluminescence, makes it suitable for plant imaging.

## 5. Conclusions

Current imaging systems comprise specialized and precision equipment such as microscopes, cameras, and computers. As a result, preparing these often becomes a bottleneck when starting new research with imaging. Smartphones have become popular as ordinary mobile devices, and annual smartphone production has exceeded 1 billion units [29]. Considering its popularity and potential, now we have the opportunity to easily use high-sensitive imaging devices. Using bioluminescence as a probe, the size of the imaging part is compact, and portability can thus be ensured. Therefore, bioluminescence could open up a new mobile imaging era.

## Supplementary Materials

The following are available online at https://www.mdpi.com/1424-8220/20/24/7166/s1, Figure S1: Structures of objective lens detachable from a smartphone, Figure S2: Accuracy evaluation of the objective lens, Figure S3: Localization of GeNL(Ca^2+^)_520-H2B in HeLa cells. Figure S4: Time-lapse bioluminescence color image of CalfluxVTN in cytosol.

## References

[1] Pawan Kalyani M. (2018). A Study of Product Differentiation Strategy in Mobile Devices Specifically In Smartphones about Smartphones Features. J. Manag. Eng. Inf. Technol..

[2] González A.B., Pozo J. (2019). The Industrial Camera Modules Market. Photonics Views.

[3] Lee S.A., Yang C. (2014). A smartphone-based chip-scale microscope using ambient illumination. Lab Chip.

[4] Hutchison J.R., Erikson R.L., Sheen A.M., Ozanich R.M., Kelly R.T. (2015). Reagent-free and portable detection of Bacillus anthracis spores using a microfluidic incubator and smartphone microscope. Analyst.

[5] Cui X., Ren L., Shan Y., Wang X., Yang Z., Li C., Xu J., Ma B. (2018). Smartphone-based rapid quantification of viable bacteria by single-cell microdroplet turbidity imaging. Analyst.

[6] Maeda M., Usuda N., Kokubo M., Shirane S., Fukasawa M., Nagayama K. (2020). A Leeuwenhoek-Type Mobile Microscope for Histology Education. Microsc. Today.

[7] Cevenini L., Calabretta M.M., Lopreside A., Tarantino G., Tassoni A., Ferri M., Roda A., Michelini E. (2016). Exploiting NanoLuc luciferase for smartphone-based bioluminescence cell biosensor for (anti)-inflammatory activity and toxicity. Anal. Bioanal. Chem..

[8] Yu H., Tan Y., Cunningham B.T. (2014). Smartphone fluorescence spectroscopy. Anal. Chem..

[9] Tran M.V., Susumu K., Medintz I.L., Algar W.R. (2019). Supraparticle Assemblies of Magnetic Nanoparticles and Quantum Dots for Selective Cell Isolation and Counting on a Smartphone-Based Imaging Platform. Anal. Chem..

[10] Sung Y., Campa F., Shih W.C. (2017). Open-source do-it-yourself multi-color fluorescence smartphone microscopy. Biomed. Opt. Express.

[11] Kuhnemund M., Wei Q., Darai E., Wang Y., Hernandez-Neuta I., Yang Z., Tseng D., Ahlford A., Mathot L., Sjoblom T. (2017). Targeted DNA sequencing and in situ mutation analysis using mobile phone microscopy. Nat. Commun..

[12] Dai B., Jiao Z., Zheng L., Bachman H., Fu Y., Wan X., Zhang Y., Huang Y., Han X., Zhao C. (2019). Colour compound lenses for a portable fluorescence microscope. Light. Sci. Appl..

[13] Hong X., Nagarajan V.K., Mugler D.H., Yu B. (2016). Smartphone microendoscopy for high resolution fluorescence imaging. J. Innov. Opt. Health Sci..

[14] Diederich B., Then P., Jugler A., Forster R., Heintzmann R. (2019). cellSTORM-Cost-effective super-resolution on a cellphone using dSTORM. PLoS ONE.

[15] Arts R., den Hartog I., Zijlema S.E., Thijssen V., van der Beelen S.H., Merkx M. (2016). Detection of Antibodies in Blood Plasma Using Bioluminescent Sensor Proteins and a Smartphone. Anal. Chem..

[16] Tomimuro K., Tenda K., Ni Y., Hiruta Y., Merkx M., Citterio D. (2020). Thread-Based Bioluminescent Sensor for Detecting Multiple Antibodies in a Single Drop of Whole Blood. ACS Sens..

[17] Suzuki K., Kimura T., Shinoda H., Bai G., Daniels M.J., Arai Y., Nakano M., Nagai T. (2016). Five colour variants of bright luminescent protein for real-time multicolour bioimaging. Nat. Commun..

[18] Hall M.P., Unch J., Binkowski B.F., Valley M.P., Butler B.L., Wood M.G., Otto P., Zimmerman K., Vidugiris G., Machleidt T. (2012). Engineered luciferase reporter from a deep sea shrimp utilizing a novel imidazopyrazinone substrate. ACS Chem. Biol..

[19] Yang J., Cumberbatch D., Centanni S., Shi S.Q., Winder D., Webb D., Johnson C.H. (2016). Coupling optogenetic stimulation with NanoLuc-based luminescence (BRET) Ca++ sensing. Nat. Commun..

[20] Hossain M.N., Suzuki K., Iwano M., Matsuda T., Nagai T. (2018). Bioluminescent Low-Affinity Ca(2+) Indicator for ER with Multicolor Calcium Imaging in Single Living Cells. ACS Chem. Biol..

[21] Weissleder R., Ntziachristos V. (2003). Shedding light onto live molecular targets. Nat. Med..

[22] Perez Koldenkova V., Nagai T. (2013). Genetically encoded Ca(2+) indicators: Properties and evaluation. Biochim. Biophys. Acta.

[23] Hattori M., Ozawa T. (2015). High-throughput Live Cell Imaging and Analysis for Temporal Reaction of G Protein-coupled Receptor Based on Split Luciferase Fragment Complementation. Anal. Sci..

[24] Nagai T., Sawano A., Park E.S., Miyawaki A. (2001). Circularly permuted green fluorescent proteins engineered to sense Ca^2+^. Proc. Natl. Acad. Sci. USA.

[25] Furukawa S., Nagamatsu A., Nenoi M., Fujimori A., Kakinuma S., Katsube T., Wang B., Tsuruoka C., Shirai T., Nakamura A.J. (2020). Space Radiation Biology for “Living in Space”. Biomed. Res. Int..

[26] da Silveira W.A., Fazelinia H., Rosenthal S.B., Laiakis E.C., Kim M.S., Meydan C., Kidane Y., Rathi K.S., Smith S.M., Stear B. (2020). Comprehensive Multi-omics Analysis Reveals Mitochondrial Stress as a Central Biological Hub for Spaceflight Impact. Cell.

[27] Gertz M.L., Chin C.R., Tomoiaga D., MacKay M., Chang C., Butler D., Afshinnekoo E., Bezdan D., Schmidt M.A., Mozsary C. (2020). Multi-omic, Single-Cell, and Biochemical Profiles of Astronauts Guide Pharmacological Strategies for Returning to Gravity. Cell Rep..

[28] Mitiouchkina T., Mishin A.S., Somermeyer L.G., Markina N.M., Chepurnyh T.V., Guglya E.B., Karataeva T.A., Palkina K.A., Shakhova E.S., Fakhranurova L.I. (2020). Plants with genetically encoded autoluminescence. Nat. Biotech..

[29] Statista. https://www.statista.com/statistics/263437/global-smartphone-sales-to-end-users-since-2007/.

