# Supplementary material for: Smartphone-Based Portable Bioluminescence Imaging System Enabling Observation at Various Scales from Whole Mouse Body to Organelle
Source: Sensors (Basel). 2020 Dec 14;20(24):7166. doi: 10.3390/s20247166 (PMC7764933; doi:10.3390/s20247166)
Supplement: Supplementary file 1 [file sensors-20-07166-s001.pdf]

**Supplemental Figures**

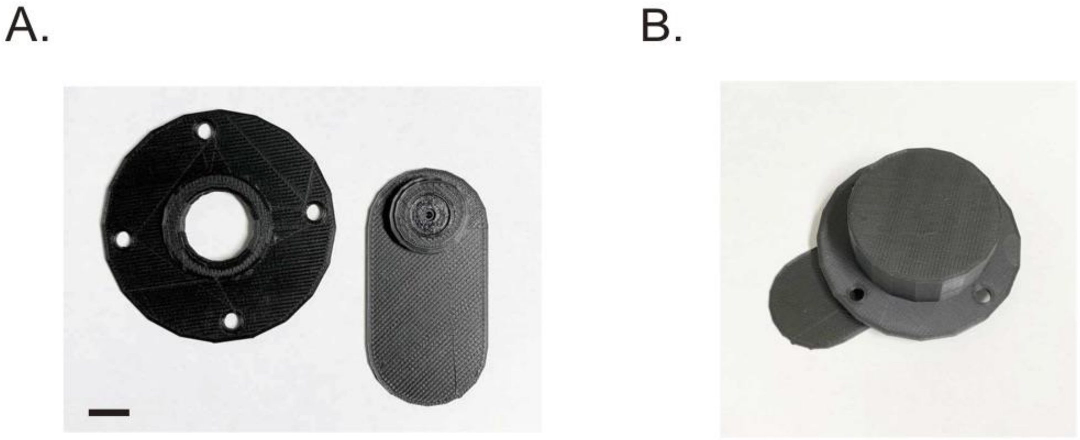


**Figure S1.** Structures of objective lens detachable from a smartphone. (A) Parts of the objective lens. The lens consists of two parts, the pedestal part and the lens part. Scale bar, 10 mm. (B) An image of a shading cover for the culture dish that blocks ambient light.

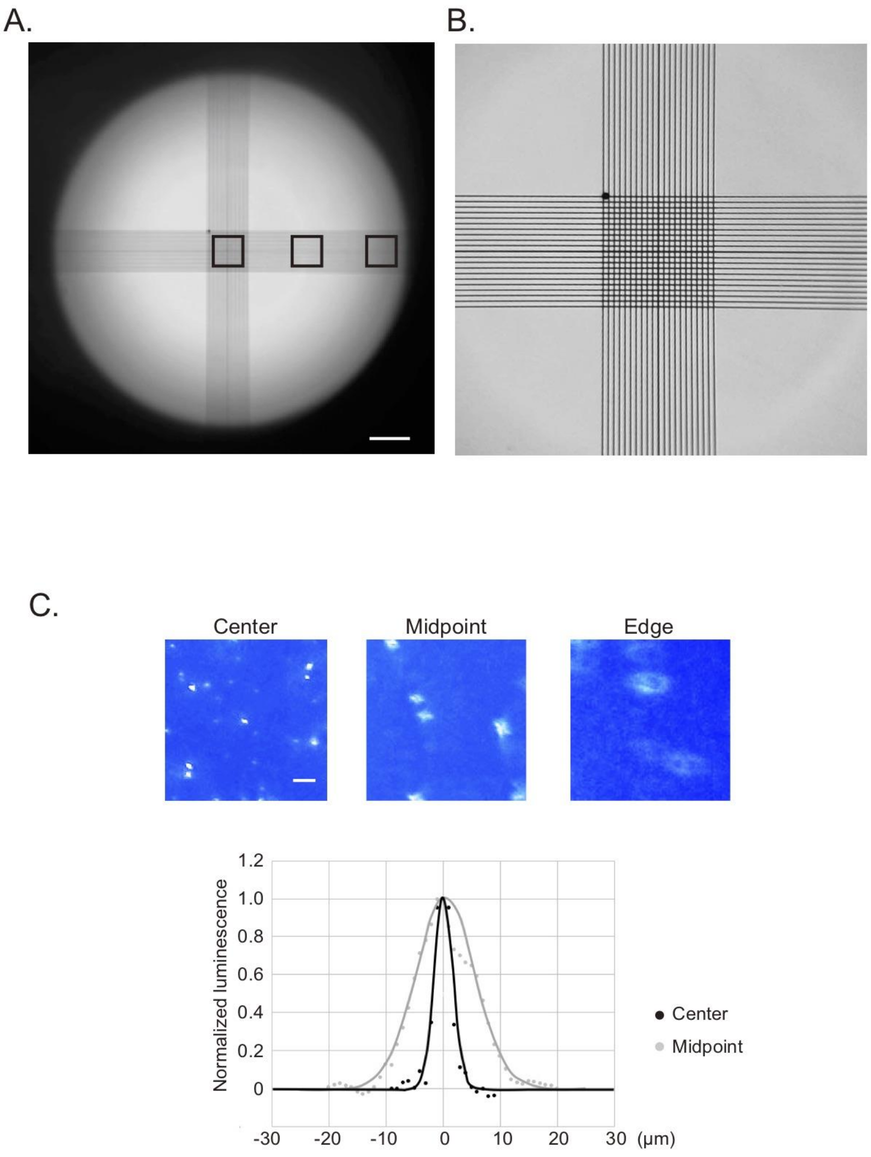


**Figure S2.** Accuracy evaluation of the objective lens. (A) A brightfield image of a micrometer taken using the smartphone microscope. Black squares indicate the selected regions shown in C. Scale bar, 1 mm. (B) Magnification of the central area of A. The minimum scale is 0.01 mm. (C) Point spread function of the lens on the microscope. Each area of the image (center, midpoint, and edge) is shown in A. Graphs were calculated by measuring the intensity of each light spot in the center and midpoint. Scale bar, 100 μm.

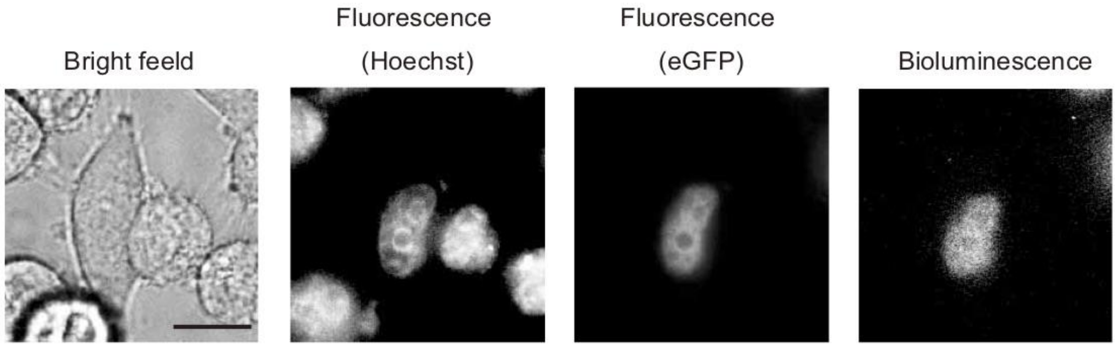


**Figure S3.** Localization of GeNL($Ca^{2+}$)_520-H2B in HeLa cells. HeLa cells transiently expressing GeNL($Ca^{2+}$)_520-H2B were cultured, and fluorescence (Hoechst and eGFP) and bioluminescence were observed using a conventional fluorescence microscope. Scale bar, 50 μm.

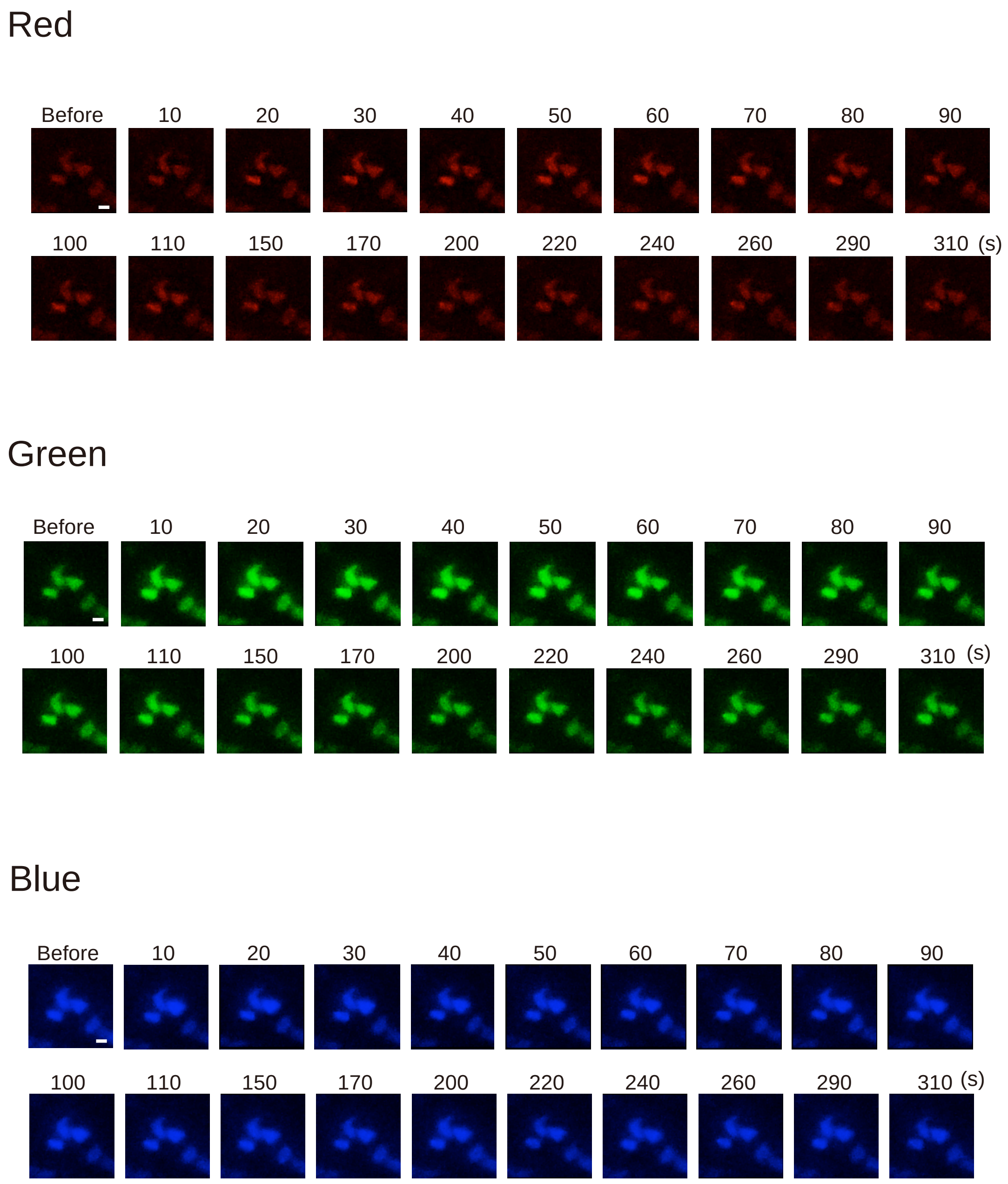


**Figure S4.** Time-lapse bioluminescence color images of CalfluxVTN in cytosol. Cells were stimulated by histamine. The numbers indicate the elapsed time after stimulation. The colors in the original images were separated into three color channels (red, green, and blue) using software. Scale bar, 20 μm.

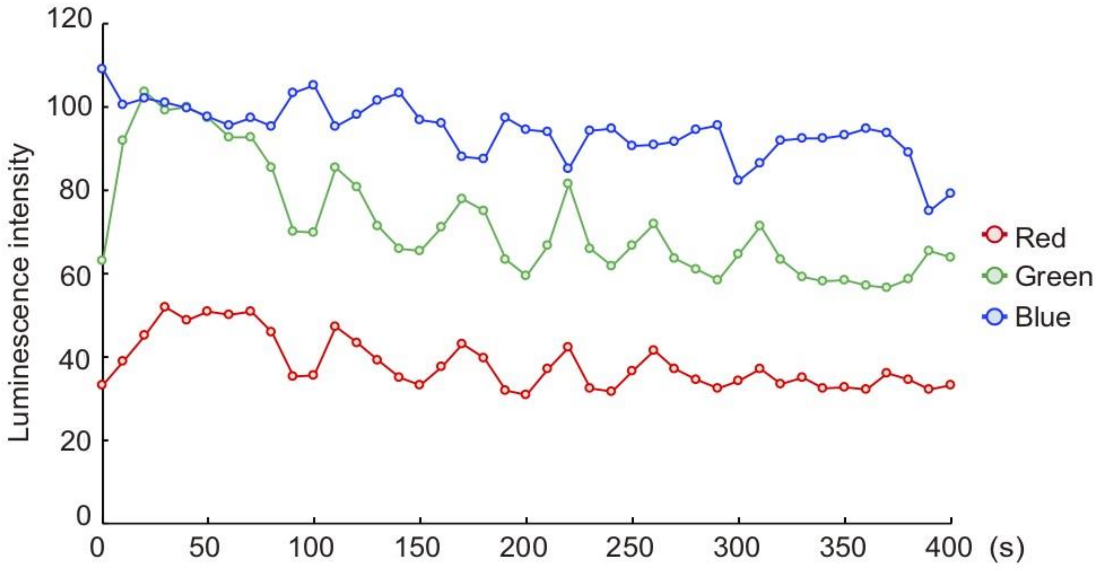


**Figure S5.** Raw data of Figure 5B. The intensity was measured for each color image separated from the original image (Figure. S4). X-axis shows the elapsed time after the stimulation (at 0).
